# Patterning nanofibrils through the templated growth of multiple modified amyloid peptides

**DOI:** 10.1038/srep31993

**Published:** 2016-08-25

**Authors:** Hiroki Sakai, Ken Watanabe, Fuki Kudoh, Rui Kamada, Yoshiro Chuman, Kazuyasu Sakaguchi

**Affiliations:** 1 Department of Chemistry, Faculty of Science, Hokkaido University, N10 W8, Kita-ku, Sapporo 060-0810, Japan

## Abstract

There has been considerable interest in the patterning of functionalized nanowires because of the potential applications of these materials to the construction of nanodevices. A variety of biomolecular building blocks containing amyloid peptides have been used to functionalize nanowires. However, the patterning of self-assembled nanowires can be challenging because of the difficulties associated with controlling the self-assembly of these functionalized building blocks. Herein, we present a versatile approach for the patterning of nanowires based on the combination of templated fibril growth with a versatile functionalization method using our structure-controllable amyloid peptides (SCAPs). Using this approach, we have succeeded in the formation of multi-type nanowires with tandem domain structures in high yields. Given that the mixing-SCAP method can lead to the formation of tandem fibrils, it is noteworthy that our method allowed us to control the initiation of fibril formation from the gold nanoparticles, which were attached to a short fibril as initiation points. This approach could be used to prepare a wide variety of fibril patterns, and therefore holds great potential for the development of novel self-assembled nanodevices.

Self-assembling molecules have been the focus of considerable research efforts because of their natural ability to spontaneously form ordered nanosized structures under mild conditions, as well as their wide range of potential applications in nanotechnology and biotechnology^1^. Functionalized nanowires are particularly attractive nanomaterials that can be produced via self-assembly. The unique one-dimensional geometry of functionalized nanowires provides them with a diverse range of unique properties, which have led to numerous applications in nanoelectronic devices and biological processes^2^,^3^,^4^.

The patterning of nanowires is a key process for the practical application of nanosized materials in nanodevices. One of the main methods for the patterning of nanowires involves the construction of a patterned functional domain structure on a single nanowire bearing two or more independent regions composed of distinct elements or molecules. This class of nanowires has been shown to possess outstanding physical properties that could allow for the realization of next-generation nanodevices^5^,^6^,^7^. Another important strategy for the patterning of nanowires involves the attachment of nanowires at specific positions on the surface of a substrate. This process is necessary for creating nanowire-based functional systems^8^,^9^. The development of a process capable of defining the initiation position of each nanowire of the surface of a substrate is highly desired because of the potential application of these materials to the interconnection of nanoelectrodes produced by nanolithography. Two groups have developed straightforward approaches for the construction of nanofibers with functional domain structures using block co-polymers^10^ or π-conjugates^11^. Several methods have also been developed for the horizontal and vertical alignment of nanofibers or locating nanofibers at specific positions on the surfaces of different substrates^12^,^13^,^14^,^15^,^16^. Jia *et al*. recently reported the preparation of uniform cylindrical polymer micelles from inorganic nanoparticles using a multistep bottom-up self-assembly approach^17^. However, no methods have been developed to date for the construction of functional domain structures or controlling the location of the fibrils on the surface of a substrate. Furthermore, there have been no reports in the literature to date pertaining to the development of methods capable of controlling the initiation position of each fibril on the surface of a substrate.

One major problem for the patterning of nanowires is the propensity of functionalized building blocks to behave in an uncontrollable manner during the self-assembly process. This lack of control can be attributed to the disruptive effects of the functional molecules attached to the nanowires^18^. Nanowire patterning processes therefore require robust conditions that are capable of controlling the self-assembly process. In this context, our previously reported method^19^,^20^, which is based on the mixing of multiple modified peptides, would provide a great opportunity for the patterning of self-assembling nanowires. These peptides contain an amyloid-forming sequence terminating in a three-amino-acid-residue unit, such as Lys_3_ or Glu_3_ at the N-terminus. Amyloid-forming peptides or amyloid peptides have long been used for the preparation of nanowires because of their capability to self-assemble into stable, highly ordered nanowire structures (amyloid fibrils)^21^,^22^. These materials are also peptidic in nature, allowing them to be readily functionalized using chemical procedures^23^,^24^,^25^. We previously reported that the nature of the three-amino-acid-residue units at the N-terminus of these peptides had a significant impact on the structure of the resulting self-assembled nanowires^19^. These modified peptides were subsequently defined as structure-controllable amyloid peptides (SCAPs). We also demonstrated that the use of Lys_3_- and Glu_3_-modified SCAPs under solution conditions is remarkably effective for the assembly of functionalized fibrils^20^. The analysis of the oligomeric state and structure of these fibrils revealed that the mixed-SCAP conditions favored the formation of heterooligomers with an extended fibril-like structure. Furthermore, these heterooligomers were not detected in the pure oligomers containing only Lys_3_- or Glu_3_-modified SCAPs. These results therefore demonstrated that the three-amino-acid-residue units in these peptides allowed them to associate with each another with appropriate binding modes during the self-assembly process, resulting in remarkable levels of control.

Herein, we show that our SCAP-mixing method is not only effective for creating functional domain structures on fibrils, but that it is also effective for defining the initiation position of a fibril on the surface of a substrate. The SCAP-mixing method was coupled to a templated growth mechanism in which the second round of successive fibril growth occurred from the ends of preformed template fibrils^26^,^27^,^28^,^29^. Two different types of SCAP mixtures were used for both rounds of growth, which allowed the formation of tandem nanowires with functional domain structures in a tandem arrangement, as shown in Fig. 1a. Statistical analysis showed that this mixing-SCAP method produced high yields of the tandem functionalized nanowires. These data therefore confirmed that the overall ratio of Lys_3_- and Glu_3_-modified SCAPs has a dramatic effect on the polarity of the templated growth, allowing for control over the geometry of the functional domain structures. We have also demonstrated that this approach can be applied to surface-immobilized gold nanoparticles to allow for the initiation position of each nanowire to be carefully defined, as shown in Fig. 1b.

## Results

### The mixing-SCAP method produced functionalized nanowires with tandem domain structures in high yields

A Lys_3_ or Glu_3_ unit was conjugated to an amyloidogenic sequence TTR(105–115)^30^,^31^, yielding two SCAPs, which will be referred to hereafter as K_3_-TTR and E_3_-TTR (all of the peptide sequences are listed in Supplementary Table S1). Carboxytetramethylrhodamine (TAMRA) and carboxyfluorescein (FAM) were used to obtain functionalized peptide building blocks. These molecules were covalently coupled to the N-termini of K_3_-TTR and E_3_-TTR, yielding TAMRA-K_3_-TTR and FAM-E_3_-TTR, respectively. These functionalized SCAPs allowed for the attached functional molecules to be readily incorporated into fibrils when they were mixed with K_3_-TTR and E_3_-TTR^20^. The fluorescent molecules attached to the fibrils also served as tracers, allowing for the detection of the tandem domain structures on the fibrils. To obtain template fibrils bearing a FAM modification, we incubated a peptide mixture of K_3_-TTR/FAM-E_3_-TTR/E_3_-TTR = 5/1/4 (overall K_3_/E_3_ = 1/1) under acidic conditions. This approach allowed for the facile construction of FAM-modified nanowires, as evidenced by the detection of green fluorescent fibrils by fluorescence microscopy (Supplementary Fig. S1a).

The formation of tandem nanowires required the removal of any free FAM-E_3_-TTR peptide from the fibril suspension. The FAM fibrils were purified by the centrifugation of the fibril suspension, followed by dialysis (see Methods for details). Fluorescence microscopy measurements indicated that no structural disruption or agglomeration occurred during the purification process (Supplementary Fig. S1b).

A peptide mixture of K_3_-TTR/TAMRA-K_3_-TTR/E_3_-TTR = 1/0.01/1 (overall ratio of K_3_/E_3_≈1/1) was then added to a suspension of the purified FAM fibrils, and the resulting mixture was incubated to a specific period. Fluorescence microscopy measurements clearly revealed green and red fluorescence at fixed locations, which were consistent with the presence of non-overlapping fluorescence domain structures on a single fibril (Fig. 2). This result therefore demonstrated the successful formation of tandem functionalized nanowires. Statistical analysis revealed a 67% yield of the tandem fibrils (Table 1). This yield was found to be remarkably high compared to those of previous studies pertaining to the formation of tandem fibrils using fluorescently modified amyloid peptides, where the majority (around 80%) of the fibrils formed spontaneously and did not possess tandem structures^27^,^29^,^32^. The fluorescence measurements also showed a very small fraction of overlapping green and red fluorescence (represented by the fibril with a yellow domain, as indicated by the blue arrow in Fig. 2c). Furthermore, all of these fibrils appeared to be wound around one or more fibrils, indicating that the overlapping fluorescence occurred as a consequence of fibril bundling. Taken together, these results highlight the efficiency of the SCAP-mixing method for the preparation of tandem functionalized nanowires. The successful formation of tandem fibrils was also supported by two control experiments. First, we prepared individual suspensions of preformed FAM and TAMRA fibrils, which were mixed and immediately observed by fluorescence microscopy. The results of this experiment showed that there were no tandem domain structures in this sample (Supplementary Fig. S2 a–c). Second, we conducted the fibrillization using a solution containing both FAM-E_3_-TTR and TAMRA-K_3_-TTR in an ensemble. This process resulted in fibrils showing a complete overlap of FAM and TAMRA fluorescence (Supplementary Fig. S2 d–f), suggesting that the coexisting functionalized SCAPs co-assembled into a fibril without forming domain structures.

### Controlling the geometry of the tandem domains by varying the overall ratio of K_3_/E_3_

The fluorescence microscopy images shown in Fig. 2c demonstrated that there were three different types of tandem fibrils, as depicted schematically in Fig. 2d, including (i) symmetric TAMRA-FAM-TAMRA (Fig. 2c orange arrowhead and Fig. 2d top); (ii) asymmetric TAMRA-FAM-TAMRA (Fig. 2c white arrowhead and Fig. 2d middle); and (iii) asymmetric TAMRA-FAM (Fig. 2c white arrow and Fig. 2d bottom). This observation was consistent with the structural polymorphology of the fibrils, in that fibrils with different degrees of growth polarity normally co-exist in an ensemble^27^,^28^. It is noteworthy, however, that statistical analysis revealed that 32% of the tandem fibrils had grown symmetrically (Table 1). This observation therefore contrasted with the strong growth polarity (i.e., rare symmetric growth) reported in various amyloid-forming peptides and proteins^27^,^28^,^33^. Taken together, these results therefore suggest that the mixed-SCAP conditions could be contributing to the symmetric fibril growth observed in the current study. To investigate this possibility in greater detail, we conducted a tandem growth experiment using SCAP mixtures with a diverse range of K_3_/E_3_ ratios. Starting with template FAM fibrils of K_3_-TTR/FAM-E_3_-TTR/E_3_-TTR = 5/1/4, the second round of growth was carried out using SCAP mixtures of K_3_-TTR/TAMRA-K_3_-TTR/E_3_-TTR = 1/0.05/9, 8/0.05/2 and 9/0.05/1 (i.e., overall K_3_/E_3_ ratios of 1/9, 8/2 and 9/1, respectively). The resulting fibrils showed different degrees of symmetric growth in the range of 28 to 46% (Table 1). These values were found to be dependent on the overall K_3_/E_3_ ratio, as evidenced by a plot of the degree of symmetric fibrils against the K_3_/E_3_ ratio (Supplementary Fig. S3). This result therefore indicates that the geometry of tandem nanowires could potentially be controlled using the SCAP-mixing method. This result represent a significant development, because of the great potential for the tandem structures of semiconductor nanowires to be used to build up highly functional nanosystems if the geometry of each domain can be highly controlled^6^,^7^. The ability of the SCAP-mixing method to allow for the facile preparation of semiconductor nanowires^20^ further highlights the significance of this approach.

It has been reported that the polarity of new growth processes can be transmitted from the polarity of the template fibrils^28^. This process can be explained by the polarity of the new growth being determined by the supramolecular structure of the template fibrils at the growing ends. Furthermore, evidence for this process has been provided by the results of several solid-state NMR studies, which revealed the presence of supramolecular structures with different arrangements of terminal β-strands at the opposing ends of the fibrils^31^,^34^. However, our results showed that, even when the same template fibrils were provided, the polarity of the new growth varied depending on the overall K_3_/E_3_ ratio of the soluble peptides. These results therefore suggest that the solution conditions could also dictate the polarity of the new growth. One possible explanation is that the oligomeric state and structure of the soluble peptides could play a major role in determining the polarity of the new growth. We previously demonstrated that solution conditions capable of yielding morphologically different fibrils also showed very different oligomeric states and structures in terms of their soluble peptides^20^. Remarkably, we also observed that the mixed-SCAP conditions induced a unique structure for the K_3_/E_3_ hetero-oligomers, which adopted an extended β-sheet-like conformation, thereby highlighting the substantial effect of the oligomeric structure on the structure of the resulting fibril. Given that the overall K_3_/E_3_ ratio can influence the ratio of K_3_/E_3_ peptides comprising a hetero-oligomer, it follows that this ratio can alter the oligomeric state and structure, leading to changes in the structure and polarity of the resulting fibrils. Further study is therefore required to address this hypothesis.

### The tandem growth of SCAP fibrils allowed for the initiation position of each fibril to be defined on the substrate surface

**Figure 1:**
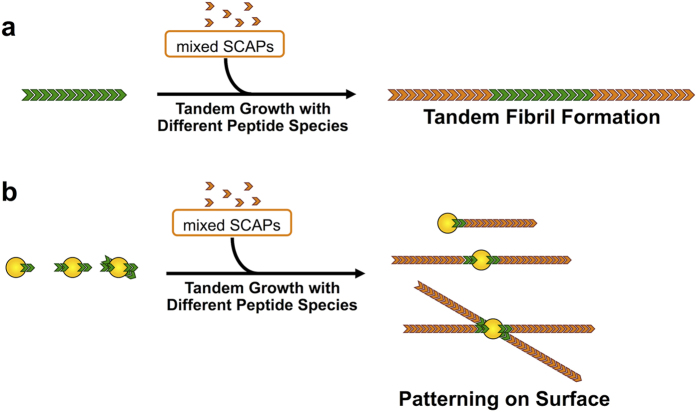
Schematic illustrations of the strategies used to create patterned nanofibers based on the tandem growth of two types of fibrils. (**a**) Secondary fibril growth (red) from a certain fibril (green) leads to a domain structure with a tandem arrangement in a single fibril. (**b**) The initiation position of the nanofibers can be controlled using the gold nanoparticles attached with the initiator fibril(s) (green).

**Figure 2:**
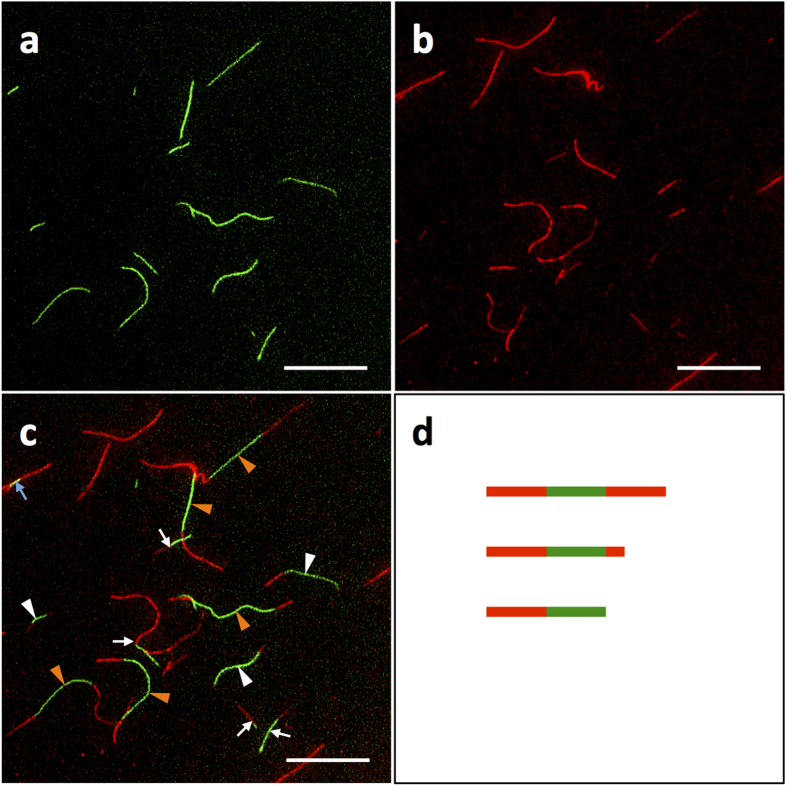
Growth of tandem fluorescent fibrils. (**a–c**) Fluorescence images of the tandem fibrils prepared by the incubation of a mixture of K_3_-TTR:TAMRA-K_3_-TTR:E_3_-TTR = 1:0.01:1 (overall K_3_:E_3_≈1:1) with purified FAM-functionalized fibrils for two days. Tandem fibrils with structures compared of symmetric TAMRA-FAM-TAMRA (orange arrowheads), asymmetric TAMRA-FAM-TAMRA (white arrowheads) and asymmetric TAMRA-FAM (white arrows) were observed. The overlapping fluorescence of TAMRA and FAM (blue arrow) was also detected but present with a very small population. (**d**) Schematic illustrations of three different types of tandem fibrils: symmetric TAMRA-FAM-TAMRA (top), asymmetric TAMRA-FAM-TAMRA (middle) and asymmetric TAMRA-FAM (bottom) fibrils. Filters with excitation/emission wavelengths of 480/535 and 540/605 nm were used to detect the FAM and TAMRA fluorescence, respectively. The scale bar represents 20 μm.

**Figure 3:**
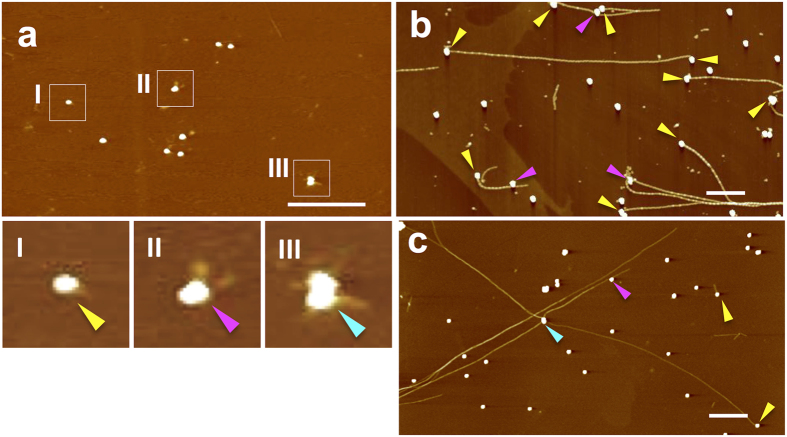
Controlling the initiation position of the fibrils. AFM images of (**a**) gold nanoparticles after the incubation of the sonicated fibrils on a mica surface (the nanoparticle-fibril complexes are indicated in white squares I–III, and are shown under digital magnification.) (**b,c**) a mica surface after the incubation of a 1/1 mixture of K_3_-TTR and E_3_-TTR with the nanoparticle-fragmented fibril complexes for 12 h (single, two-branched, and four-branched fibrils initiated from a single nanoparticle are indicated with yellow, magenta, and cyan arrowheads, respectively.). The scale bars represent 1 μm.

Template fibrils with molecular recognition properties can bind to small nanoobjects on the surface of a substrate, allowing these nanoobjects to act as initiation points for the second growth phase of the fibrils (Fig. 1b). For targeting gold nanoparticles as initiation points, we prepared α-lipoylated fibrils with the ability to bind to gold using the SCAP-mixing method with an α-lipoylated SCAP (Supplementary Fig. S4a). The resulting α-lipoylated fibrils were then sonicated into small fragments (Supplementary Fig. S4b). Well-dispersed, single gold nanoparticles were deposited on a mica surface, which was pre-coated with poly-L-lysine (PLL) to block the non-specific electrostatic adsorption of the fibrils onto the mica^12^. The fragmented α-lipoylated fibrils were then incubated with the surface-immobilized gold nanoparticles. AFM measurements of the surface allowed for the visualization of the fragmented fibrils bound to the gold nanoparticles (magnified areas I–III in Fig. 3a). There were patterns of the fragmented fibril-bound gold nanoparticles, including one fragment per one gold nanoparticle (I) and multiple fragments per gold nanoparticle (II, III). The surface was subsequently exposed to a solution of K_3_-TTR/E_3_-TTR = 1/1 mixture, which allowed for the growth of the fibrils from single gold nanoparticles. As shown in Fig. 3b,c and Supplementary Fig. S5, single and branched fibrils from the gold nanoparticles were formed. Some of the fibrils even connected two gold nanoparticles.

The number of fibrils initiated from the nanoparticles corresponded to 75% of the total number of fibrils (*N* = 81). Only a small proportion of the fibrils grew from the particles in a symmetrical manner (~5%), most likely because one end of the template fibrils was blocked or had become unfavorable for tandem growth because of the binding of the gold nanoparticles. It is also possible that the growth conditions (K_3_/E_3_ = 1/1) favored the growth of asymmetric tandem fibrils (Table 1). No fibril formation was observed when the peptide mixture was incubated with gold nanoparticles alone on mica (Supplementary Fig. S6), thereby highlighting the importance of the α-lipoylated fibril fragments being attached to the nanoparticles. These results therefore demonstrate that the templated growth of SCAP fibrils targeting gold nanoparticles is a useful approach for controlling the location of each nanowire on the surface of a substrate. To the best of our knowledge, this work represents the reported first account of a method capable of controlling the initiation position of “single” peptide fibrils on the surface of a substrate.

## Discussion

In summary, we have a new approach for patterning self-assembling nanowires based on the coupling of a templated fibril growth mechanism with a SCAP-mixing method. This approach produced tandem functionalized nanowires in high yields. Furthermore, we were able to control the geometrical characteristics of these tandem nanowires by varying the overall ratio of K_3_/E_3_ units introduced to the soluble peptides. Our approach also allowed for each fibril to grow from individual gold nanoparticles present on the surface of a substrate. The ability of the SCAP-mixing method to yield diverse functionalized fibrils^20^ was critical to the success of this approach because it allowed for a variety of functional molecules to be incorporated into template fibrils, as well as growing the fibrils. This process could be used to achieve diverse functionalization patterns on tandem nanowires as well as the specific targeting of various nanoobjects as initiation points. Patterning methods capable of branched tandem nanowires from gold nanoparticles are promising methods for the creation of patterned nanostructures as next-generation nanodevices^5^,^6^. This method would be compatible with the gold nanoobjects produced using lithographic processes. Our SCAP-based approach could therefore be used for integrating self-assembled functionalized nanowires into nanocircuitry, creating new opportunities for the construction of novel nanowire-based devices.

## Methods

### Materials

All of the peptides were synthesized using standard Fmoc-chemistry and identified as described previously^19^,^20^. N-Terminal modifications were conducted on the resins and the resulting peptides were obtained in ≥95% purity. Citrate-stabilized gold nanoparticles with a diameter of 40 nm were purchased from Tanaka Kikinzoku Kogyo (Tokyo, Japan). The ligand of the nanoparticles was exchanged to bis(*p*-sulfonatophenyl)phenylphosphine (BSPP, Wako Pure Chemical Industries, Osaka, Japan) prior to the experiments. The citrate-stabilized nanoparticles were initially centrifuged at 14,000 *×g* for 15 min at rt. The supernatant was removed and the nanoparticles were treated with an aqueous solution of BSPP (1 mg/ml) and incubated overnight at rt. The nanoparticles were then washed twice using the same procedure. Fibril formation was conducted as described previously^19^,^20^. Briefly, 8 mM stock solutions of each peptide mixture were dissolved in 1,1,1,3,3,3-hexafluoro-2-propanol, and the resulting mixtures were diluted 40-fold in water at pH 2.0 (adjusted by HCl) before being incubated at 37 °C.

### Fibril purification

A 60 μl suspension of preformed mature fibrils (K_3_-TTR/FAM-E_3_-TTR/E_3_-TTR = 5/1/4) was mounted on 60 μl of a sucrose solution (62.5% w/v) in a centrifuge tube, and the resulting mixture was centrifuged at 43,000 *×g* for 30 min at 20 °C. Forty microliters of the supernatant was removed and 80 μl of the residual solution was dialyzed with water (pH 2.0) using a 10 k MWCO dialyzer (Slide-A-Lyzer Mini Dialysis Units, Thermo Fisher Scientific, Waltham, MA, USA).

### Tandem growth of TAMRA fibrils from the template FAM fibrils

A 175 μl suspension of purified FAM fibril was mixed with 5 μl of the peptide stock solutions (8 mM in HFIP) and 8 μl of TAMRA-K_3_-TTR (25 μM in pH 2.0 water), and the resulting mixture was diluted with water (pH 2.0) to a total volume of 200 μl. Mixtures of the purified FAM fibrils and the soluble peptides of K_3_-TTR/TAMRA-K_3_-TTR/E_3_-TTR = 1/0.05/9, 5/0.05/5 (1/0.01/1), 8/0.05/2 and 9/0.05/1 (overall K_3_/E_3_ ratio of 1/9, 1/1, 8/2, and 9/1, respectively) were incubated at 37 °C for 0.5, 2, 4 and 8 days, respectively, which represent the times required for the new fibrils to reach several micrometers in length. Samples for fluorescence microscopy were prepared as described previously^20^.

### Growth of tandem fibrils from gold nanoparticles

The mica surface was pre-coated with 10 μl of a 1 mg/ml solution of PLL (M_W_ = 15–30 k, Sigma, St. Louis, MO, USA) for 1 min at rt, before being washed three times with 1 ml of ultra-pure water and dried in air. Ten microliters of BSPP-stabilized gold nanoparticles (4.5 × 10^−4 ^wt%) were subsequently incubated for 5 min and the surfaces of the nanoparticles were then washed and dried in a similar manner to that described above. PLL was used again according to the same procedure. A suspension of sonicated α-lipoyl fibrils (30 min with W-113 MK-II ultrasonic disperser, Honda Electronics, Aichi, Japan) was diluted 3-fold, and a 5 μl sample of the resulting mixture was incubated for 5 min at rt. The surface of the fibrils was then washed and dried. For the incubation of the peptides, a double-stacked silicone chamber (CultureWell multiwell chambered coverslip, 3 mm diameter and 1 mm depth, Grace Bio-Labs, Bend, OR, USA) was mounted on the surface of the fibrils. Eight microliters of a peptide solution of K_3_-TTR/E_3_-TTR = 1/1 at a total peptide concentration of 200 μM was incubated in the chamber at 37 °C for 12 h, during which time the top was sealed with a glass cover. The surface was subsequently washed and dried in air.

### Microscopy

AFM and fluorescence microscopy measurements were performed as described previously^19^,^20^ with a Nanoscope IIIa system (Degital Instruments, Santa Barbara, CA) or a Nanocute (Hitachi High-Tech Science Corp. Japan) using Si cantilever (SI-DF20, Hitachi High-Tech Science Corp. Japan).

## Additional Information

**How to cite this article**: Sakai, H. *et al*. Patterning nanofibrils through the templated growth of multiple modified amyloid peptides. *Sci. Rep.* **6**, 31993; doi: 10.1038/srep31993 (2016).

## Supplementary Material

Supplementary Information

## Figures and Tables

**Table 1:** Statistical analysis of the fibrils counted after the second fibril growth.

	K_3_/E_3_ = 1/9	K_3_/E_3_ = 1/1	K_3_/E_3_ = 8/2	K_3_/E_3_ = 9/1
Symmetric TAMRA-FAM-TAMRA	6.8 ± 2.3	21.4 ± 2.8	21.8 ± 1.8	32.9 ± 4.0
Asymmetric TAMRA-FAM-TAMRA	5.7 ± 2.7	13.1 ± 0.3	7.0 ± 0.3	10.6 ± 0.3
Asymmetric TAMRA-FAM	11.5 ± 5.5	32.6 ± 4.5	30.6 ± 4.5	28.5 ± 7.1
TAMRA	71.8 ± 4.8	28.5 ± 1.5	34.7 ± 4.1	17.9 ± 2.6
FAM	4.2 ± 2.4	4.4 ± 0.8	6.0 ± 2.5	10.1 ± 0.2
Total tandem yield^a^	24.0 ± 4.6	67.2 ± 1.9	59.3 ± 6.6	72.0 ± 2.9
Degree of symmetric tandem fibrils^b^	28.3 ± 10.2	31.9 ± 5.2	36.7 ± 1.0	45.7 ± 7.3

^a^Total number of tandem fibrils over the total number of fibrils.

^b^Total number of symmetric TAMRA-FAM-TAMRA fibrils over the total number of tandem fibrils.

Peptide mixtures of K_3_-TTR/TAMRA-K_3_-TTR/E_3_-TTR = 1/0.05/9, 5/0.05/5 (1/0.01/1), 8/0.05/2 and 9/0.05/1 (overall K_3_/E_3_ ratios of 1/9, 1/1, 8/2 and 9/1, respectively) were used. All of these values represent percentages for *N* = 383, 411, 386 and 368 fibrils, respectively. The errors represent the standard deviations.
