# Supplementary material for: Patterning nanofibrils through the templated growth of multiple modified amyloid peptides
Source: Sci Rep. 2016 Aug 25;6:31993. doi: 10.1038/srep31993 (PMC4997568; doi:10.1038/srep31993)
Supplement: Supplementary Information [file srep31993-s1.pdf]

Supplementary Information

Patterning nanowires through the templated growth of multiple modified amyloid peptides

Hiroki Sakai, Ken Watanabe, Fuki Kudoh, Rui Kamada, Yoshiro Chuman & Kazuyasu Sakaguchi[1,*]

Department of Chemistry, Faculty of Science, Hokkaido University, N10, W8, Kita-ku, Sapporo 060-0810, Japan

*E-mail: kazuyasu@sci.hokudai.ac.jp, TEL: +81-11-706-2698, FAX: +81-11-706-4683

Contents

Table S1. The peptide sequences used in this study

Figure S1. Fluorescence microscopy images of fluorescein-functionalized fibrils

Figure S2. Fluorescence microscopy images of control fluorescent fibrils

Figure S3. K_3/E_3-ratio dependency of the degree of symmetric tandem fibrils

Figure S4. AFM image of fragmented α-lipoylated fibrils

Figure S5. AFM image of the nanoparticle-fibril complexes

Figure S6. AFM image of a control experiment for the initiation of fibrils from a gold nanoparticle

Table S1. Peptide sequences used in this study. All of the peptides were synthesized using standard Fmoc solid-phase chemistry.

Peptide	Sequence
E_3-TTR	H-EEE-GG-YTIAALLSPYS-NH_2
K_3-TTR	H-KKK-GG-YTIAALLSPYS-NH_2
FAM-E_3-TTR	5,6-carboxyfluorescein-GG-EEE-GG-YTIAALLSPYS-NH_2
TAMRA-K_3-TTR	5,6-carboxytetramethylrhodamine-GG-EEE-GG-YTIAALLSPYS-NH_2
Lip-E_3-TTR	α-lypoyl-EEE-GG-YTIAALLSPYS-NH_2

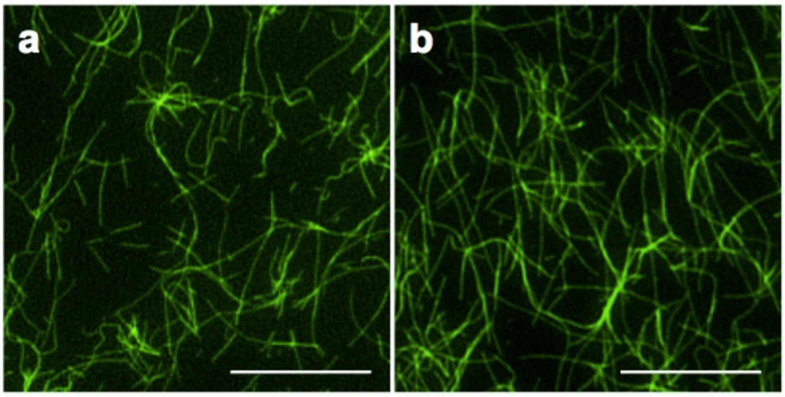

Figure S1. Fluorescence microscopy images of the fluorescein-functionalized fibrils formed from a mixture of K_3-TTR:FAM-E_3-TTR:E_3-TTR before (a) and after (b) purification via centrifugation and dialysis. The scale bar represents 20 μm.

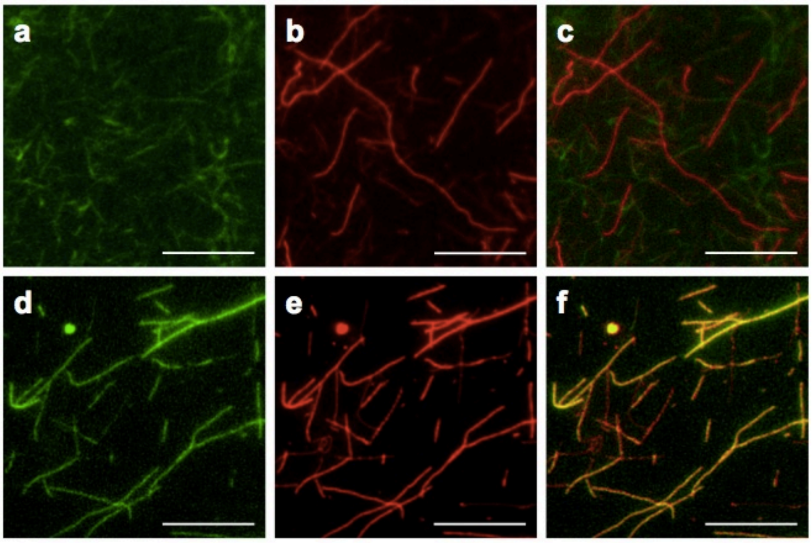

Figure S2. (a–c) Mixing of individually formed FAM and TAMRA fibrils, and (d–f) co-fibrillation with FAM-E_3-TTR and TAMRA-K_3-TTR peptides did not lead to the formation of tandem structures on the fibrils (c,f). Excitation/emission wavelengths were 480/535 and 540/605 nm in (a,d) and (b,e), respectively, and each image was superimposed (c,f). The scale bar represents 20 μm.

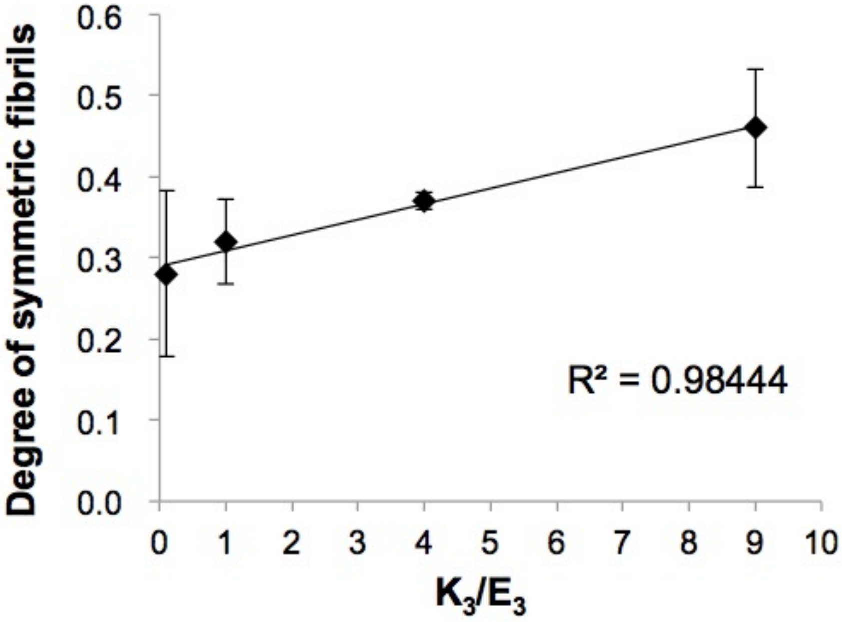

Figure S3. K_3/E_3-ratio dependency of the degree of symmetric tandem fibrils. The line represents the linear regression with R^2 of 0.988. The error bars represent standard deviations.

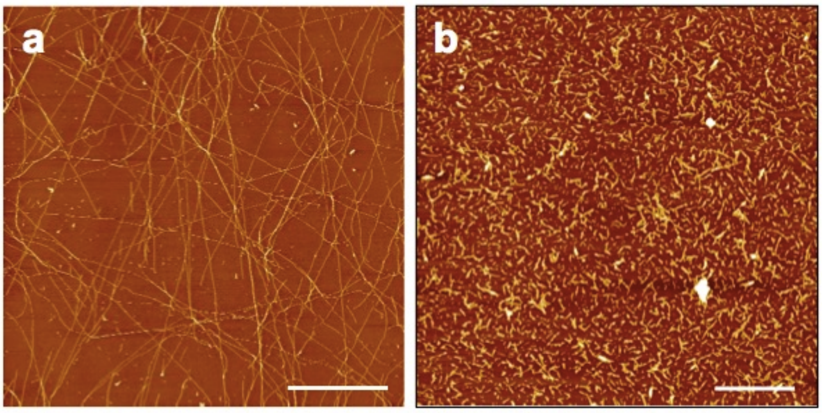

Figure S4. AFM images of the α-lipoylaged fibrils (a) before and (b) after sonication. The fibrils were adsorbed on mica. The average length of the sonicated fibrils was 45±20 nm. The scale bars represent 5 and 1 μm in (a) and (b), respectively.

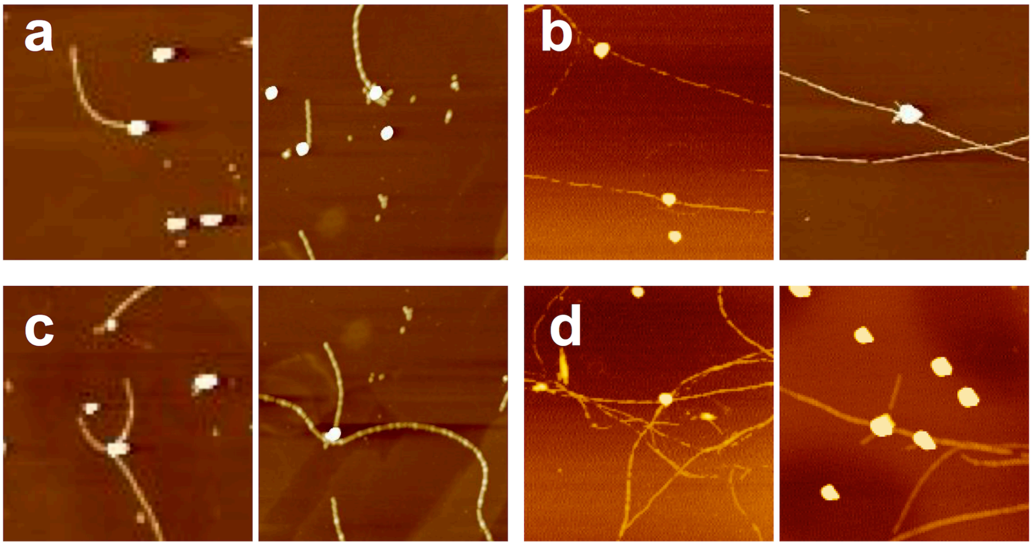

Figure S5. AFM image of the nanoparticle-fibril complexes. One fibril per one gold nanoparticle (**a**), two fibrils (**b**), three fibrils (**c**), and four fibrils (**d**) per one gold nanoparticle were shown.

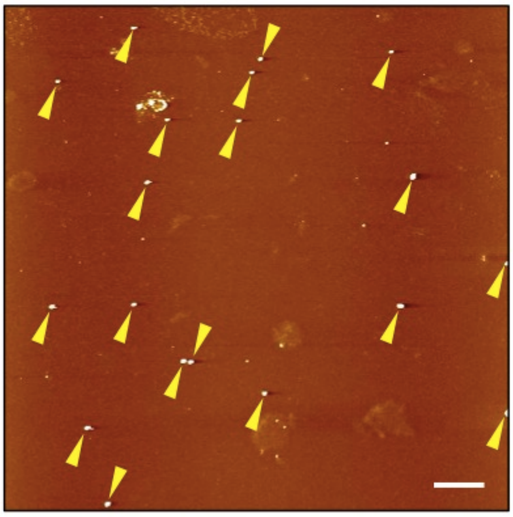

Figure S6. AFM image of a mica surface after the incubation of a mixture of K_3-TTR:E_3-TTR = 1:1 with fibril fragment-free gold nanoparticles. The nanoparticles are indicated by the yellow arrowheads. The scale bar represents 1 μm.
